# Etiology and antibiotic susceptibility of bacterial pathogens responsible for community-acquired urinary tract infections in Poland

**DOI:** 10.1007/s10096-016-2673-1

**Published:** 2016-05-18

**Authors:** E. Stefaniuk, U. Suchocka, K. Bosacka, W. Hryniewicz

**Affiliations:** Department of Epidemiology and Clinical Microbiology, National Medicines Institute, Chełmska 30/34, 00-725 Warsaw, Poland; Faculty of Agriculture and Biology, Warsaw University of Life Sciences, Nowoursynowska 166, 02-787 Warsaw, Poland; Military Preventive Medicine Center in Cracow, Odrowąża 7, 30-901 Cracow, Poland; Centre of Quality Control in Microbiology, Rydygiera 8 (20A), 01-793 Warsaw, Poland

## Abstract

Urinary tract infections (UTIs) are some of the most common infections in both community and hospital settings infections. With their high rate of incidence, recurrence, complications, diverse etiologic agents, as well as growing antibiotic resistance, UTIs have proven to be a serious challenge for medical professionals. The aim of this study was to obtain data on the susceptibility patterns of pathogens responsible for UTIs in Poland to currently used antibiotics. A total of 396 bacterial isolates were collected between March and May 2013 from 41 centers in all regions of Poland. The majority of isolates were from adult patients (96.2 %); 144 (37.8 %) patients were diagnosed with uncomplicated UTI, while the remaining 237 (62.2 %) had a complicated infection. The most prevalent pathogen was *Escherichia coli* (71.4 %), followed by *Klebsiella* spp. (10.8 %) and the Proteae group (7.6 %). *Escherichia coli* was responsible for 80.6 % of cases of uncomplicated and 65.8 % of complicated infections. Only 65.8 % of *E. coli* isolates were susceptible to ciprofloxacin (uncomplicated 75.9 %, complicated 58.3 %), 64.0 % to nitrofurantoin (67.2 %, 62.8 %), 65.1 % to trimethoprim/sulfamethoxazole (68.1 %, 62.8 %), and 66.4 % to fosfomycin (77.6 %, 62.2 %). Among *E. coli* isolates from all UTIs, only 43.4 % were susceptible to ampicillin, with 47.4 % from uncomplicated compared with 40.4 % from complicated infections; 88.2 % to amoxicillin/clavulanic acid (91.4 % vs. 85.9 % complicated); 90.1 % to cefuroxime (93.1 %, 87.8 %); and 94.1 % to cefotaxime (98.2 %, 91.0 %). Thirty-five strains (10.4 %) were capable of producing extended-spectrum β-lactamases (ESBLs). This study demonstrates an increase in multidrug-resistant strains, especially among the leading pathogens associated with UTIs, including *E. coli*, *Klebsiella* spp., and *Proteus* spp.

## Introduction

Urinary tract infections (UTIs) are some of the most common infections in both community and hospital settings. With their high rate of incidence, recurrence, complications, diverse etiologic agents, as well as growing antibiotic resistance, UTIs have proven to be a serious challenge for medical professionals. They account for 10–20 % of all infections treated in primary care and 30–40 % of infections treated in hospitals.

Rapidly increasing bacterial resistance to antibiotics resulting in significantly limited treatment options and multidrug resistance is a growing concern. Therefore, knowledge of the current antibiotic susceptibility patterns of the major bacterial pathogens responsible for UTIs is important in order to permit the optimal choice for empiric treatment. The aim of this study is to provide the most up-to-date data on the epidemiology, etiology, and susceptibility patterns of major pathogens responsible for community-acquired UTIs in Poland. In addition, clinically important mechanisms of resistance and risk factors in patients predisposing to UTIs were analyzed.

## Materials and methods

The study was performed in the Department of Epidemiology and Clinical Microbiology (DECM) of the National Medicines Institute in Warsaw on a total of 396 bacterial isolates collected between March and May 2013 from 41 centers, representing all regions of Poland. The isolates were recovered from ambulatory patients with clinical symptoms of UTI and significant bacteriuria [1, 2]. One isolate per patient was included in the study. The patients’ demographic and epidemiological data were collected and analyzed.

### Identification

All strains were reidentified by using ID GP and ID GN cards in VITEK Compact (bioMérieux, Marcy l’Etoile, France) and stored at −70 °C in TSB broth supplemented (with blood for streptococci) and 10 % glycerol. Before performing further studies, the strains were unfrozen and subcultured twice on Columbia agar plates with 5 % sheep blood (Bio-Rad, Marnes-la-Coquette, France).

### Antimicrobial susceptibility

Susceptibility to a range of antibiotics was evaluated by the disk diffusion method on Mueller–Hinton agar (MHA; Bio-Rad, Marnes-la-Coquette, France) for Gram-negative rods, staphylococci, and enterococci, and MHA supplemented with 5 % defibrinated horse blood and 20 mg/L NAD (Bio-Rad, Marnes-la-Coquette, France) for streptococci. Reading of the disk diffusion susceptibility results was performed automatically using the ADAGIO apparatus (Bio-Rad, Marnes-la-Coquette, France).

The following disks for susceptibility testing were used: ampicillin (10 μg), amoxicillin/clavulanic acid (20 μg/10 μg), amikacin (30 μg), benzylpenicillin (1 U), cefalexin (30 μg), cefepime (30 μg), cefotaxime (30 μg), ceftazidime (30 μg), cefuroxime (30 μg), ciprofloxacin (5 μg), erythromycin (15 μg), gentamicin (10 μg), gentamicin (30 μg), imipenem (10 μg), clindamycin (2 μg), meropenem (10 μg), nitrofurantoin (100 μg), piperacillin/tazobactam (100 μg/10 μg), teicoplanin (30 μg), trimethoprim/sulfamethoxazole (11.25 μg/23.75 μg), fosfomycin (200 μg), vancomycin (5 μg) (Bio-Rad, Marnes-la-Coquette, France). Minimum inhibitory concentrations (MICs) were determined by VITEK Compact (bioMérieux, Marcy l’Etoile, France) and, in addition, by antibiotic gradient strips, Etest (bioMérieux, Marcy l’Etoile, France), and MICE (Oxoid Limited, Hampshire, United Kingdom), for extended-spectrum β-lactamase (ESBL)-producing strains.

The interpretation of susceptibility results was performed according to the European Committee on Antimicrobial Susceptibility Testing (EUCAST) guidelines [3], with the exception of fosfomycin, which was interpreted according to the Clinical and Laboratory Standards Institute (CLSI) guidelines [4].

ESBL production was detected by the double-disk synergy test (DDST) [5]. To detect genes encoding ESBLs, specific primers were used for polymerase chain reaction (PCR) assays. β-Lactamases were profiled by isoelectrofocusing, as described previously [6–8]. Total bacterial DNA was purified with a Genomic DNA Prep Plus kit (A&A Biotechnology, Gdansk, Poland). PCR products were subjected to electrophoresis in a 1 % agarose gel (Blirt, Gdansk, Poland) with ethidium bromide (Merck, Darmstadt, Germany).

High-level aminoglycoside resistance (HLAR) detection in enterococci was performed by disk diffusion with a 30-μg gentamicin disk (Bio-Rad, Marnes-la-Coquette, France) [3].

### Quality control

*Escherichia coli* ATCC 25922, *E. coli* ATCC 35218, *Enterococcus faecalis* ATCC 29212, *Klebsiella pneumoniae* ATCC 700603, *Pseudomonas aeruginosa* ATCC 27853, and *Streptococcus pneumoniae* ATCC 49619 from the collection of the National Medicines Institute in Warsaw were used as reference strains.

## Results

The majority of isolates were from adult patients (*n* = 381; 96.2 %) aged 19 to 94 years (average 60 years), with patients aged less than 40 years (*n* = 67) accounting for 17.6 % of the total. As there were only 15 isolates (3.8 %) from children aged 1–18 years, these were excluded from further analysis. Among isolates from adults, more than 77.2 % (*n* = 294) were derived from females, and females predominated in all age groups. The majority of strains (*n* = 207; 54.3 %) were derived from patients aged 65 years and over, of which 150 (72.5 %) were from females and 57 (27.5 %) were from males. One hundred and forty-four (37.8 %) patients were diagnosed with uncomplicated UTI, while the remaining 237 (62.2 %) had a complicated infection. The latter group comprised patients presenting with recurrent UTI (*n* = 126; 58.3 %), urinary/fecal incontinence (*n* = 67; 31.0 %), nephritis (*n* = 51; 23.6 %), kidney stones (*n* = 46, 21.3 %), prostate hypertrophy (*n* = 12; 5.56 %), renal insufficiency (*n* = 7; 3.2 %), diabetes (*n* = 9; 4.17 %), kidney empyema (*n* = 4; 1.85 %), polycystic kidneys (*n* = 3; 1.39 %), and kidney cancer (*n* = 3; 1.39 %).

Aerobic Gram-negative bacteria were the dominant pathogens (*n* = 356; 93.4 %). The majority (*n* = 347; 91.1 %) of isolates belonged to the Enterobacteriaceae, while 9 (2.5 %) strains belonged to the non-fermenting rods. The most frequently isolated species from both uncomplicated and complicated infections were *E. coli* (71.4 %), followed by *Klebsiella* spp. (10.8 %) and the Proteae group (7.6 %), including *Proteus mirabilis*, *P. vulgaris*, *Providencia rettgeri*, and *Morganella morganii* in uncomplicated UTI. *Escherichia coli* was responsible for 80.6 % of cases, compared with 65.8 % of complicated cases. The proportion of *Klebsiella* spp. and *Proteus* spp. causing complicated UTIs was higher compared with those causing uncomplicated infections, accounting for 13.5 % vs. 6.3 % and 9.7 % vs. 3.5 %, respectively. *Escherichia coli* was mainly responsible for UTIs in females (*n* = 229; 77.9 %), followed by *Klebsiella* spp. (*n* = 26; 8.8 %) and *Proteus* spp. (*n* = 13; 4.4 %). In females aged less than 65 years, *E. coli* accounted for 86.7 % of uncomplicated and 74.7 % of complicated infections (*p* = 0.34). Among females aged 65 years and over, *E. coli* was responsible for 72.1 % of uncomplicated and 68.5 % of complicated infections. It is interesting that, among females aged less than 30 years (*n* = 36), *E. coli* and *E. faecalis* were responsible for 88.9 % and 11.1 % of UTIs, respectively, with no other pathogen isolated.

Almost half of the isolates from UTI in men were *E. coli* (*n* = 43; 49.4 %), followed by *Klebsiella* spp. (*n* = 15; 17.2 %). A higher proportion of *Proteus* spp. was observed in men compared to women, with 16.1 % and 4.4 %, respectively (*p* = 0.01).

Susceptibility to antimicrobial agents was higher among isolates from uncomplicated than complicated infections (Table 1). Among *E. coli* isolates from all UTIs, only 43.4 % were susceptible to ampicillin, with 47.4 % from uncomplicated compared with 40.4 % from complicated infections; 88.2 % to amoxicillin/clavulanic acid (91.4 % uncomplicated, 85.9 % complicated); 90.1 % to cefuroxime (93.1 %, 87.8 %); and 94.1 % to cefotaxime (98.2 %, 91.0 %). Only 65.8 % of *E. coli* isolates were susceptible to ciprofloxacin (uncomplicated 75.9 %, complicated 58.3 %), 64.0 % to nitrofurantoin (67.2 %, 62.8 %), 65.1 % to trimethoprim/sulfamethoxazole (68.1 %, 62.8 %), and 66.4 % to fosfomycin (77.6 %, 62.2 %). Resistance to three or more drugs was higher among isolates recovered from complicated than uncomplicated infections (41.0 % vs. 13.8 %). The most common resistance phenotype comprised resistance to ampicillin, ciprofloxacin, and trimethoprim/sulfamethoxazole. Over 90 % of *E. coli* isolates recovered from both types of infections were susceptible to aminoglycosides, third- and fourth-generation cephalosporins, and piperacillin/tazobactam, and all were susceptible to carbapenems.

**Table 1:** Susceptibility of all Enterobacteriaceae rods and *Escherichia coli* isolated from uncomplicated and complicated community-acquired urinary tract infections (UTIs) to various antimicrobial agents

Antimicrobials	All cases of UTIs (*N* = 381)		Uncomplicated UTIs (*N* = 144)		Complicated UTIs (*N* = 237)	
	Enterobacteriaceae strains (*n* = 347)^a^	*E. coli* (*n* = 272)	Enterobacteriaceae strains (*n* = 133)^b^	*E. coli* (*n* = 116)	Enterobacteriaceae strains (*n* = 214)^c^	*E. coli* (*n* = 156)
	Susceptible strains (n’; %)					
	n’ (%)	n’ (%)	n’ (%)	n’ (%)	n’ (%)	n’ (%)
Ampicillin	134 (38.6 %)	118 (43.4 %)	58 (43.6 %)	55 (47.4 %)	76 (35.5 %)	63 (40.4 %)
Amoxicillin/clavulanic acid	295 (84.0 %)	240 (88.2 %)	120 (90.2 %)	106 (91.4 %)	175 (81.8 %)	134 (85.9 %)
Piperacillin/tazobactam	310 (89.3 %)	253 (93.0 %)	131 (98.5 %)	115 (99.1 %)	179 (83.6 %)	138 (88.5 %)
Cefuroxime	285 (82.1 %)	245 (90.1 %)	120 (90.2 %)	108 (93.1 %)	165 (77.1 %)	137 (87.8 %)
Cefotaxime	300 (86.5 %)	256 (94.1 %)	128 (96.2 %)	114 (98.2 %)	172 (80.4 %)	142 (91.0 %)
Ceftazidime	306 (88.2 %)	259 (95.2 %)	127 (95.5 %)	114 (98.2 %)	179 (83.6 %)	145 (93.0 %)
Cefixime	310 (89.3 %)	260 (95.6 %)	128 (96.2 %)	114 (98.2 %)	183 (85.5 %)	146 (93.6 %)
Cefepime	316 (91.1 %)	264 (97.1 %)	129 (94.9 %)	115 (99.1 %)	187 (87.4 %)	149 (95.5 %)
Imipenem	344 (99.1 %)	272 (100 %)	131 (98.5 %)	116 (100 %)	213 (99.5 %)	156 (100 %)
Meropenem	347 (100 %)	272 (100 %)	133 (100 %)	116 (100 %)	214 (100 %)	156 (100 %)
Amikacin	333 (96.0 %)	269 (98.9 %)	132 (99.3 %)	116 (100 %)	201 (93.9 %)	153 (98.1 %)
Gentamicin	308 (88.8 %)	252 (92.7 %)	124 (93.2 %)	110 (94.8 %)	184 (86.0 %)	142 (91.0 %)
Ciprofloxacin	211 (60.8 %)	179 (65.8 %)	98 (73.7 %)	97 (75.9 %)	113 (52.8 %)	91 (58.3 %)
Nitrofurantoin	194 (55.9 %)	174 (64.0 %)	81 (60.9 %)	88 (67.2 %)	113 (52.8 %)	98 (62.8 %)
Fosfomycin	nd	188 (66.4 %)	nd	90 (77.6 %)	nd	97 (62.2 %)
Trimethoprim/sulfamethoxazole	209 (60.2 %)	177 (65.1 %)	89 (66.9 %)	79 (68.1 %)	120 (56.1 %)	98 (62.8 %)

nd, not determined

^a^Enterobacteriaceae strains responsible for UTIs: *E. coli* (*n* = 272), *K. pneumoniae* (*n* = 39), *K. oxytoca* (*n* = 2), *P. mirabilis* (*n* = 25), *P. vulgaris* (*n* = 2), *P. rettgeri* (*n* = 1), *C. freundii* (*n* = 2), *C. koseri* (*n* = 1), *E. cloacae* (*n* = 1), *S. marcescens* (*n* = 1), *M. morganii* (*n* = 1)

^b^Enterobacteriaceae strains responsible for uncomplicated UTIs: *E. coli* (*n* = 116), *K. pneumoniae* (*n* = 9), *P. mirabilis* (*n* = 5), *C. freundii* (*n* = 1), *C. koseri* (*n* = 1), *M. morganii* (*n* = 1)

^c^Enterobacteriaceae strains responsible for complicated UTIs: *E. coli* (*n* = 156), *K. pneumoniae* (*n* = 30), *K. oxytoca* (*n* = 2), *P. mirabilis* (*n* = 20), *P. vulgaris* (*n* = 2), *P. rettgeri* (*n* = 1), *C. freundii* (*n* = 1), *E. cloacae* (*n* = 1), *S. marcescens* (*n* = 1)

In women aged 65 years or more with uncomplicated infections, susceptibility of *E. coli* to ciprofloxacin and trimethoprim/sulfamethoxazole was lower than in those aged less than 65 years (88.9 % vs. 54.6 % and 76.4 % vs. 54.6 %, respectively). In addition, differences were observed in the susceptibility of *E. coli* to trimethoprim/sulfamethoxazole and ciprofloxacin recovered from complicated infections in men versus women (68.1 % vs. 48.8 % and 62.8 % vs. 46.5 %, respectively).

Production of ESBLs was found in 2.8 % of Enterobacteriaceae recovered from uncomplicated infections compared with 13.5 % of isolates from complicated infections. *Klebsiella pneumoniae* was the pathogen most frequently associated with ESBL production, with 46.2 % of all *K. pneumoniae* isolates being positive.

All ESBLs belonged to the CTX-M family and were classified on the basis of their reaction with specific primers, with 35 isolates belonging to CTX-M-1 and one to CTX-M-9.

Only nine non-fermenting Gram-negative rods were identified: seven *Pseudomonas aeruginosa* and two *Acinetobacter baumannii*. These were all recovered from patients with complicated UTI. *Pseudomonas aeruginosa* isolates were broadly susceptible to the key antibiotics tested, with 100 % susceptibility to carbapenems, ciprofloxacin, piperacillin/tazobactam, and gentamicin. One of the *A. baumannii* strains was resistant to carbapenems.

Gram-positive cocci were isolated from 25 patients and accounted for 6.6 % of all the isolates, with 20 *E. faecalis* and five *S. agalactiae* identified. The former were most frequently recovered from females (*n* = 13), while *S. agalactiae* were isolated exclusively from females. In 48 % of cases, both of these pathogens were responsible for complicated infections. The *E. faecalis* isolates were fully susceptible to penicillin, ampicillin, nitrofurantoin, fosfomycin, and vancomycin; however, almost 35 % presented with the HLAR phenotype. *Streptococcus agalactiae* was fully susceptible to the antibiotics tested.

## Discussion

The data presented show that *E. coli* remains the most common etiologic agent of community-acquired UTI in Poland, although its role in etiology differs depending on the type of infection (uncomplicated vs. complicated) and the patient’s characteristics. The overall percentage of *E. coli* as an etiologic agent of community-acquired UTIs in Poland decreased from 83.7 % in our previous study published in 1998/99 to 71.4 % in 2013 [9]. However, *E. coli* still predominates in uncomplicated UTIs (80.6 %) and even more so in younger females aged less than 30 years (88.9 %). A multicenter study (ARESC) on uncomplicated UTIs carried out in nine European countries and Brazil showed that *E. coli* was responsible for 76.7 % of infections, ranging from 68.1 % in Austria to 83.8 % in France [10]. Among Polish isolates included in this study, *E. coli* was responsible for 75.6 %, which is somewhat lower than that revealed in the present investigation.

In this study, we observed a decreasing percentage of *E. coli* among the Enterobacteriaceae responsible for complicated infections. In particular, we noted an increase in *Klebsiella* spp. from 2.8 % in 1998/99 to 10.8 % in the current study, mainly in complicated infections, resulting in an increase in ESBL-positive strains and, thus, multidrug-resistant isolates. By contrast, the percentage of *Proteus* spp. did not change significantly compared with the previous study [9]. Similar distributions of etiological agents causing UTI have been presented by other authors [10–17]. In the current study, over 46 % of *Klebsiella* spp. were found to produce ESBLs and all but one of these isolates were recovered from complicated infections and all but one were found to belong to CTX-M group 1, which is the most common ESBL type in Poland [18]. A high prevalence of ESBL-producing Enterobacteriaceae has also been demonstrated in other studies [11, 15, 19]. A recent publication also indicates that ESBL-producing strains are more frequent in older patients [20], which is similar to the findings presented here.

From analyses of uncomplicated infections in the ARESC study [10], which included data from Poland, only a low percentage (5.6 %) of *K. pneumoniae* isolates producing ESBLs were identified, and this is comparable to the results obtained in the current paper. In our study, *Proteus* spp. was particularly frequent in males, accounting for more than 16.1 % of cases compared with 5.3 % in females, which was also reported in other publications [17, 20].

In general, Gram-positive pathogens are much less frequently isolated from UTIs than the Enterobacteriaceae and were responsible for 6.6 % of all infections in this study and 5 % in our previous study [9]. Similar data are also published from other countries [15, 21, 22]. In the present study, only *E. faecalis* and *S. agalactiae* were recovered, with no single isolate of *S. saprophyticus*. The lack of the isolation of the latter species may result from the fact that they are mainly responsible for uncomplicated infections in young women, who constituted only 19 % (*n* = 56) of our collection. This group of patients is usually given empiric treatment without taking a sample for culture [10, 16, 21, 23, 24]. Another reason for the lack of *S. saprophyticus* in our study collection could be due to the time of the year over which the study was conducted, i.e., winter, as it was previously observed in a previous study from Europe and the US that the prevalence of this pathogen varies depending on the time of the year observed [10, 21, 25]. In the ARESC study, *S. saprophyticus* was found to be responsible for only 4.2 % of all cases of uncomplicated UTI [26].

Several recent studies have underlined an important change in resistance patterns among urinary pathogens. Since *E. coli* is the leading cause of UTI, an increase in resistance of this pathogen is of particular concern. Although the proportion of ESBL-producers among this pathogen and the other members of Enterobacteriaceae has not increased compared to our earlier study, the proportion of resistance to other antibiotics has increased substantially. The most notable changes among *E. coli* causing uncomplicated UTI cases have been observed for nitrofurantoin, ciprofloxacin, trimethoprim/sulfamethoxazole, and fosfomycin, with susceptibility to nitrofurantoin decreasing to 67.2 % (from 91.2 % in the previous study), susceptibility to ciprofloxacin decreasing to 73.7 % (from 96.1 %), susceptibility to trimethoprim/sulfamethoxazole decreasing to 68.1 % (from 80.9 %), and susceptibility to fosfomycin decreasing to 77.6 % (from 96.7 %) [9]. Additionally, important differences in susceptibility were noticed between *E. coli* isolated from younger (aged less than 65 years) and older (aged 65 years and more) females in a study of uncomplicated infections, especially in terms of susceptibility to ciprofloxacin (88.8 % vs. 54.5 %) and trimethoprim/sulfamethoxazole (76.4 % vs. 54.6 %). Similar results have been reported in a recent paper from Hong Kong [27]. This may be due to the fact that older females usually have more recurrent infections that were previously treated and could have had more contact with hospital settings.

Two recent studies have underlined that *E. coli* is isolated less frequently from males than from females and that isolates from complicated infections in males are much more resistant [17, 28]. Our study confirmed this finding. The percentage of isolates susceptible to ciprofloxacin in *E. coli* strains from females and males were 62 % and 46.5 %, respectively, and to trimethoprim/sulfamethoxazole were 68.1 % and 48.8 %, respectively. Nitrofurantoin is one of the most frequently used drugs for treating uncomplicated UTIs, and resistance to this drug is generally very low, as indicated by numerous studies [29–31], including earlier data from Poland [9, 10, 22, 26]. A high proportion of resistance to this drug among *E. coli* as presented in this study is of concern. The only explanation for this finding could be the very high consumption of furazidin, a member of the same chemical group as nitrofurantoin, in Poland. This drug has been available without prescription in Poland since 2011. The sales have been increasing steadily year-on-year and its consumption as measured in defined daily doses (DDD) per 1000 population per day has increased from 2.13 in 2010 to 2.83 in 2012, an increase of 32.9 %. Looking at the susceptibility data of the isolates from complicated UTIs examined in this study, the treatment options for oral therapy are very limited, since less than 60 % of *E. coli* were susceptible to ciprofloxacin and slightly over 63 % were susceptible to trimethoprim/sulfamethoxazole.

The increasing resistance among bacterial populations of urinary tract pathogens is particularly indicated by the growing number of multidrug-resistant strains. Multidrug resistance is defined as non-susceptibility to at least one agent in each of three or more antimicrobial groups [32]. Important differences between countries and regions with regard to the percentage of MDR among *E. coli* UTI isolates have been reported, ranging from 16.7 % in Portugal to 34 % in California, and as high as 76.5 % in India [29, 33, 34]. In our study, multidrug-resistant *E. coli* isolated from complicated infections was found to be 41 %. The most common risk factors associated with multidrug-resistant *E. coli*, identified in almost 21 % of patients in our study, were nephritis and other renal pathologies, while in a study from India, diabetes was determined to be one of the most common risk factors for multidrug resistant *E. coli*, in addition to renal pathologies [34].

Multidrug resistance is the most rapidly increasing resistance phenotype, particularly among *K. pneumoniae*, due to the efficient spread of ESBL-producing, and more recently carbapenemase-producing, strains [35–38]. Currently, such strains are mostly isolated from hospitalized patients, but their presence is now increasing rapidly in the community. In our study, 71.8 % of *K. pneumoniae* presented with the multidrug resistance phenotype, with the most common resistance pattern including resistance to cefuroxime, ceftazidime, ciprofloxacin, and trimethoprim/sulfamethoxazole. Ongoing monitoring of resistance trends is of great importance.

## Conclusion

This study presents the changing etiology of urinary tract infections (UTIs) in Poland over the past 14 years and demonstrates an increase in multidrug-resistant strains, especially among the leading pathogens associated with UTIs, including *Escherichia coli*, *Klebsiella* spp., and *Proteus* spp. It also highlights the importance of ongoing surveillance at the local level in order to guide empiric treatment recommendations for local use.
